# Maturity model for assessing the medical humanities: a Delphi study

**DOI:** 10.1186/s12909-024-05356-8

**Published:** 2024-04-03

**Authors:** Xin Zhang, Zhiguang Duan

**Affiliations:** School of Mangement, Shanxi Medical University, 030001 TaiYuan, China

**Keywords:** Medical humanities, Maturity model, Delphi, First-level discipline

## Abstract

**Background:**

Becoming a first-level discipline in China means access to more educational resources. The development of medical humanities in China has been going on for more than 40 years, and some medical schools have set up master’s and doctoral programs in medical humanities. The demand for medical humanities-related knowledge in China is also growing after COVID-19. However, medical humanities is only a second-level discipline and receives limited resources to meet the needs of society. This study aims to establish a system of indicators that can assess whether the medical humanities has a first-level discipline and provide a basis for its upgrading to a first-level.

**Methods:**

A Delphi technique was used, with the panel of expert expressing their views in a series of two questionnaires. A coefficient of variation of less than 0.2 indicates expert agreement.

**Result:**

A total of 25 experts participated in this Delphi study. Consensus was reached on 11 first-grade indices and 48 s-grade indices. The authoritative coefficient(Cr) of the experts was 0.804, which indicates that the experts have a high level of reliability.

**Conclusion:**

This study provides a reliable foundation for the evaluation of medical humanities maturity.

## Introduction

William Osler introduced the concept of the “medical humanist” in 1919 [1]. He believed that a good medical worker should combine the love of humanity with the love of technology in the practice of medicine, to realize the trinity of heart, body, and mind. George Sarton put forward the concept of “medical humanities” in 1947 [2]. George Sarton proposed the concept of “medical humanities” in 1947, calling for the establishment of “new humanities” that would complement scientific knowledge and reposition the significance of existing scientific discoveries and inventions for humanity and culture. In recent years, the connotation of “medical humanities” has become richer and richer, but scholars have not yet agreed on its concept. In recent years, the connotation of “medical humanities” has become richer and richer, but scholars have not yet agreed on its concept [3]. At the theoretical level, medical humanities is a cross-disciplinary subject that brings together knowledge from various disciplines such as medicine, history, ethics, philosophy, and psychology. At the practical level, medical humanities is a series of movements with the same purpose, methodology, and participant groups for understanding and curing diseases at a deeper level [4].

Medical humanities has been developed for over 100 years, and has experienced three waves of development: advocating liberal arts, focusing on bioethics, and reflecting on medicine [5]. Since its inception, the medical humanities have had a tremendous mission: to soften the rough edges of medicine, harmonize the relationship between technology and the humanities, and mend the emotional rift brought about by biomedicine [6]. As society develops, issues such as homosexuality, reproductive rights, abortion, and gene editing require medical humanities solutions [7, 8]. A practical, concrete and complete theory is needed to support it. Medical humanities in China began in the 1980s. Currently, most of the country’s medical schools offer medical humanities programs [9]. Some medical universities even have master’s and doctoral programs in medical humanities. However, there is not yet a unified disciplinary affiliation of the various medical humanities programs [10]. The high demand for medical humanities contrasts with the “marginalization” of medical humanities.

### Overview of discipline evaluation in China

The establishment of disciplines in China is based on the relevant documents issued by the Academic Degrees Committee of the State Council and the Ministry of Education of the People’s Republic of China [11]. At present, a discipline system has been formed that includes three levels of discipline categories, first-level disciplines, and second-level disciplines [12]. Whether or not it is a first-level discipline determines the resources received in enrollment, education, and research [13]. China has carried out five rounds of discipline assessment for first-level disciplines in 1995, 2002, 2006, 2012, and 2020. Assessment is lacking for secondary and tertiary disciplines [14]. However, the second-level discipline has never been assessed and has only been studied qualitatively by scholars. In China, medical humanities is categorized as a second-level discipline under the first-level of medicine, management and education.

### Maturity model

Establishing a medical humanities maturity model (MHMM) against the criteria for a first-level discipline can provide a basis for medical humanities to apply to become a first-level discipline. Maturity models, also known as growth stage models and stage theory models, focus on constructing a baseline and are used to determine an organization’s current capabilities and future improvements [15]. The maturity model is defined as the logical path of an organization from an initial state to a mature state [16]. There are three main types of maturity models: process-based maturity models, capability-based maturity models, and hybrid maturity models [17]. Most current maturity models evolved from the Capability Maturity Model (CMM) model, which was developed in 1986 by the U.S. Department of Defense in collaboration with Carnegie Mellon University [18]. The CMM model is divided into five maturity levels: initial, repeatable, defined, managed, and optimized. The CMM model is also an essential reference for MHMM modeling.

### Life cycle theory

The model of the accumulation of knowledge is a dominant model of scientific development, which considers the development of science as a knowledge-accumulating activity [19]. Disciplinary Life Cycle Theory is a typical model of the accumulation of knowledge [20]. According to the life cycle theory, the disciplinary maturity model can be categorized into germination, development, maturity, and decline [21]. In the germination, the research are emerging, relying on scientific research projects to train talents, and a formal organization has not yet been established. During the developmental period, knowledge production accelerated, specialized journals appeared, and organizational structures were established. In maturity, the theoretical system of the discipline is perfected, and academic organizations branch out.

The objective of this study is to establish a model suitable for assessing disciplines that have not yet become first-level disciplines based on first-level disciplinary assessment criteria and to evaluate the maturity of the medical humanities discipline to promote the development of medical humanities education in China.

## Methods

### Study design

This study was designed according to a modified Delphi method [22]. Between June 2023 and August 2023, 2 rounds of Delphi surveys were conducted by QR code.

### Expert panel selection

The expert panel for this study consisted of Chinese experts from provincial degree committees, university faculty, and professional associations. All experts have more than five years of experience. The threshold requirements for the title and education of experts are an intermediate title and a Bachelor’s degree.

### Delphi procedure

This study first designed the preliminary disciplinary assessment indexes based on the conditions for the establishment of first-level disciplines published by the Chinese Ministry of Education. Disciplinary assessment indicators include (a)Research object; (b)Research object; (c)Knowledge base; (d)Research methods; (e)The secondary discipline; (f)Degree granting; (g)Talent cultivation; (h)Scientific research; (i)Course system; (j)Teachers; (k)Social need. Secondly, the development cycle of a discipline is categorized into germination, development, maturity, and decline according to the life cycle theory. Thirdly, it lists the reference markers for disciplinary assessment indicators at germination, development, maturity, and decline. (Table 1) In addition, the questionnaire included demographic information of the experts on the occupation, age, title, and education.

**Table 1:** Disciplinary maturity assessment index and reference markers within each cycle

Indicators	Characteristics of different stages
Research object (a)	1 Field X focuses on a variety of specific problems of the same type
	2 Field X focuses on different types of problems
	3 Several types of problems studied in the field of X share a common property
	4 Field X studies a class of problems with well-defined attributes, and the attributes of these problems are shifting
Theoretical system (b)	1 Field X uses a few authoritative scholarly studies as a source of theory
	2 Researchers in the field of X have summarized, generalized, and developed different theoretical results from their own practice
	3 The field of X has developed a well-established theoretical system recognized by the academic community
	4 The academic community in the field of X continuously adjusts and improves its theoretical system according to the different contexts of the times
Knowledge base (c)	1 The basic knowledge of field X has been disseminated by only a few researchers.
	2 Field X dissemination of knowledge through academic journals, books and other vehicles
	3 Knowledge dissemination through various vehicles such as teaching materials, books, audio and video in field X
	4 Knowledge dissemination through various vehicles such as teaching materials, books, audio and video in field X
Research methods (d)	1 Research methods in field X are mainly qualitative and relatively homogenous
	2 Research methods in field X are beginning to focus on the use of quantitative methods, and methods are beginning to diversify
	3 A mix of qualitative and quantitative research methods has become the norm in research in field X, and there is a discipline-appropriate, scientific approach to research
	4 A mix of qualitative and quantitative research methods has become the norm in research in field X, and there is a discipline-appropriate, scientific approach to research
The secondary discipline (e)	1 Field X has research on multiple unsettled problems and no stable scholarly outputs
	2 There are consistent and stable academic outputs in a number of research directions in field X, but there is still no established disciplinary orientation
	3 Field X has developed several self-contained and relatively fixed disciplinary directions, and each direction has sustained and stable academic outputs
	4 On the basis of the original disciplinary directions, a number of new research directions have been added to the X-field, and these directions differ from the original research directions in terms of research targets
Degree granting (f)	1 No institution has yet established a master’s degree or doctorate in X
	2 A few institutions offer master’s and doctoral programs in X fields
	3 Most institutions offer master’s and doctoral programs in X fields
	4 Institutions with master’s and doctoral programs in X are on the decline
Talent cultivation (g)	1–1 There are currently no master’s or doctoral students in X field in the community
	2–1 A certain number of master’s and doctoral students have graduated in X field
	3 − 1 There are a relatively large number of master’s and doctoral students enrolled in X field
	4 − 1 Decrease in the number of students enrolled in master’s and doctoral programs in field X
	1–2 There are only a few research grants in field X
	2–2 Funding for research in field X is beginning to increase, but it does not match the training of master’s and doctoral students
	3 − 2 Adequate funding for research in field X to enable quality training of master’s and doctoral students
	4 − 2 Funding for research in field X began to decline
Scientific research (h)	1 Scientific research in field X is still in its infancy and the results are limited
	2 X field currently has a certain number of national or provincial important academic programs, and academic achi
	3 Field X currently has a high number of important national or provincial academic programs and a proliferation of academic achievements
	4 Significant academic programs in field X are beginning to decline, with a tendency to shift in other directions
Course system (i)	1 The X-field program focuses on academic lectures by leading researchers
	2 There are instructional programs for graduate and doctoral students in Area X
	3 Field X has a systematic curriculum, and most institutions offer required and elective courses in field X
	4 Shift in curriculum content in field X
Teachers (j)	1 Only a few researchers in field X are disseminating relevant knowledge
	2 There is a critical mass of faculty in field X that can sustain relevant teaching assignments
	3 Field X has a faculty with a reasonable knowledge structure, age structure, and structure of professional and technical positions, and can consistently teach at a high level
	4 There is a tendency for teachers in field X to move to other disciplines
Social needs (k)	1 Society is not yet aware of the need for professionals in field X
	2 There is a social demand for people in field X
	3 Society has a steady and sizeable demand for talent in X field
	4 Society’s demand for talent in field X has declined

1 germination, 2 development, 3 maturity, 4 decline

In phase 1 of the survey, the authors asked the panelists to rate the importance of the assessment indices and the rationality of each assessment index across the life cycle reference markers. Panelists were also asked to rate the expert judgment (according to practical experience, theoretical analysis, domestic and international data, and intuition) and familiarity. (Table 2) They also asked them to provide any feedback or comments.

**Table 2:** The basis for expert evaluation

The basis of your judgment on the above indicators and the extent of their impacts						
Basis of judgment	Large		Medium		Small	
Practical experience	0.5		0.4		0.3	
Theoretical analysis	0.3		0.2		0.1	
domestic and international data	0.1		0.1		0.1	
Intuition	0.1		0.1		0.1	
	Your familiarity with the content of the survey					
Familiarity	Extremely	Very		Somewhat	Slightly	Not at all
Experts in their own words	5	4		3	2	1

After phase 1 of the survey, the panel adjusted the indices on which there was no consensus based on expert opinion. Panelists were asked to complete the revised questionnaire as they did in phase 1 to seek a consensus index.

### Determining consensus

Minimizing the variance around the outcome is the ultimate goal for establishing consensus [23]. The mean, standard deviation (SD), and coefficient of variation (CV) were calculated for all scores in both phases of the survey. The CV < 0.2 was used as a threshold for the indicator to enter the disciplinary maturity assessment.

### Data collection and analysis

Questionnaire were distributed and collected on WeChat in the form of a QR code. All results were imported into EXCEL (Microsoft Excel version 365) for organization. The mean and standard deviation of all indices were calculated using SPSS 26.0.

CV shows the degree of relative concentration of opinions among panelists, CV = SD/Mean. Lower CV values indicate a higher concentration of panelists. The authoritative coefficient (Cr) is composed of the judgment (Ca) and the expert’s familiarity with the problem (Cs). The formula is Cr = (Ca + Cs)/2. Cr>0.7 is an acceptable level.

## Results

### Indicator setting

a. Research object.

Hegel once said, “As far as the object is concerned, every discipline begins with the study of two questions: first, this object exists. Secondly, what is this object?” The object of study of a discipline begins with an objective entity but is not equivalent to it; it is the thinking and cognition of the subject of study about the object, not the object itself [24]. The emergence of a new discipline is bound to be the emergence of phenomena or objects that cannot be explained by existing disciplines. The emergence of the same type of phenomenon or object will gather a group of researchers with the same interest to form an academic community in the field, and these researchers will produce a continuous and stable doctrine of the field. The existence of object crossover between different fields or the unity of research purpose will trigger the macro thinking and cognition of researchers on the object, which will eventually form the research object of a discipline. Thus the description of the problem of the object of study of the discipline at different times is proposed:

Emergence (1): Field X focuses on a variety of specific problems of the same type.

Developmental (2): Domain X focuses on different types of problems.

Maturity (3): Domain X studies several types of problems that share a common set of attributes.

Decline (4): Domain X studies a class of problems with well-defined attributes, and the attributes of these problems are undergoing a transformation.

b. Theoretical system.

Hull divides the formation of concepts into three stages: abstraction, analogy, and discrimination. Abstraction: abstraction of various features and attributes of concrete things [25]. Classification: Individuals categorize the various attributes of objective things and their characteristics. Discrimination: the individual recognizes the differences between the attributes or characteristics of objective things. Theoretical systems all belong to the research subject’s abstract knowledge of objective entities, so their formation also follows the three processes of concept formation, of which abstraction and analogization should correspond to the developmental and maturity stages.

1 Field X uses a few authoritative scholarly studies as a source of theory.

2 Researchers in the field of X have summarized, generalized, and developed different theoretical results from their own practice.

3 The field of X has developed a well-established theoretical system recognized by the academic community.

4 The academic community in the field of X continuously adjusts and improves its theoretical system according to the different contexts of the times.

c. Knowledge base.

Knowledge base is the sum of various elements of knowledge involved in a subject area. Nonaka and Takeuchi categorize knowledge into tacit and explicit [26]. Explicit knowledge, also known as coded knowledge, is something that can be passed on and learned through certain carriers. Tacit knowledge, also known as tacit knowledge, exists only in the experience and feelings of individuals. According to the theory of the disciplinary development cycle, the development process of the knowledge base of disciplines should be the process of knowledge transformation from tacit knowledge to explicit knowledge.

1 The basic knowledge of field X has been disseminated by only a few researchers.

2 Field X dissemination of knowledge through academic journals, books and other vehicles.

3 Knowledge dissemination through various vehicles such as teaching materials, books, audio and video in field X.

4 Knowledge dissemination through various vehicles such as teaching materials, books, audio and video in field X.

d. Research methods.

At the early stage of the formation of a discipline, things are understood through analysis, synthesis, generalization, and reasoning based on personal feelings. During the formation period of the discipline, the research method will be influenced by philosophy and other disciplines. During the formative period of the discipline, natural science research methods characterized by positivism are promoted, and the methods gradually tend to be diversified.

1 Research methods in field X are mainly qualitative and relatively homogenous.

2 Research methods in field X are beginning to focus on the use of quantitative methods, and methods are beginning to diversify.

3 A mix of qualitative and quantitative research methods has become the norm in research in field X, and there is a discipline-appropriate, scientific approach to research.

4 A mix of qualitative and quantitative research methods has become the norm in research in field X, and there is a discipline-appropriate, scientific approach to research.

e. The secondary discipline.

The knowledge of a new discipline should be in the form of dots in its infancy. In the developmental stage, it exists in the form of fan lines. In the maturity phase, it exists in the form of a tree. The points represent the sporadic problems in the discipline, the fan line represents the research direction connected by the points, and the tree represents a perfect discipline system.

1 Field X has research on multiple unsettled problems and no stable scholarly outputs.

2 There are consistent and stable academic outputs in a number of research directions in field X, but there is still no established disciplinary orientation.

3 Field X has developed several self-contained and relatively fixed disciplinary directions, and each direction has sustained and stable academic outputs.

4 On the basis of the original disciplinary directions, a number of new research directions have been added to the X-field, and these directions differ from the original research directions in terms of research targets.

f. Degree granting.

The number of institutions offering master’s and doctoral degrees in related disciplines is used as a marker for different periods of development.

1 No institution has yet established a master’s degree or doctorate in X.

2 A few institutions offer master’s and doctoral programs in X fields.

3 Most institutions offer master’s and doctoral programs in X fields.

4 Institutions with master’s and doctoral programs in X are on the decline.

g. Talent cultivation.

The number of master’s and doctoral students and research funding are used as markers of talent training efforts in different development periods.

1–1 There are currently no master’s or doctoral students in X field in the community.

2–1 A certain number of master’s and doctoral students have graduated in X field.

3 − 1 There are a relatively large number of master’s and doctoral students enrolled in X field.

4 − 1 Decrease in the number of students enrolled in master’s and doctoral programs in field X.

1–2 There are only a few research grants in field X.

2–2 Funding for research in field X is beginning to increase, but it does not match the training of master’s and doctoral students.

3 − 2 Adequate funding for research in field X to enable quality training of master’s and doctoral students.

4 − 2 Funding for research in field X began to decline.

h. Scientific research.

The quantity and quality of scientific research projects are used as markers of scientific research at different periods of development.

1 Scientific research in field X is still in its infancy and the results are limited.

2 X field currently has a certain number of national or provincial important academic programs, and academic achi.

3 Field X currently has a high number of important national or provincial academic programs and a proliferation of academic achievements.

4 Significant academic programs in field X are beginning to decline, with a tendency to shift in other directions.

i. Course system.

Use the type of curriculum system as a marker of curriculum systems at different periods of development.

1 The X-field program focuses on academic lectures by leading researchers.

2 There are instructional programs for graduate and doctoral students in Area X.

3 Field X has a systematic curriculum, and most institutions offer required and elective courses in field X.

4 Shift in curriculum content in field X.

j. Teachers.

Use the number of teachers, age structure, etc. as an indication of the faculty at different periods of development.

1 Only a few researchers in field X are disseminating relevant knowledge.

2 There is a critical mass of faculty in field X that can sustain relevant teaching assignments.

3 Field X has a faculty with a reasonable knowledge structure, age structure, and structure of professional and technical positions, and can consistently teach at a high level.

4 There is a tendency for teachers in field X to move to other disciplines.

k. Social needs.

To take the social demand for relevant professionals as a sign of the development of the social context at different times.

1 Society is not yet aware of the need for professionals in field X.

2 There is a social demand for people in field X.

3 Society has a steady and sizeable demand for talent in X field.

4 Society’s demand for talent in field X has declined.

### The expert panel

The expert panel consisted of 25 members, including three staff members of the Provincial Degree Committee, eight faculty members specializing in medical humanities, two staff members of the Medical Humanities Association, and 14 members of the medical staff. About the age of the experts, one was under 30 years old, 11 were 30–40 years old, nine were 40–50 years old, and four were over 50 years old. Concerning the titles of the experts, 4 are professors, 11 are associate professors, and 10 are lecturers. About the educational qualifications of the experts, 11 were Ph.D. holders, 13 were master’s degree holders, and one was a bachelor’s degree holder.

### Phase 1

During the first phase, the response rate of the experts was 100%(25/25). The mean CV for indices importance was 0.129, and the mean CV for indices descriptive accuracy across cycles was 0.153. For the 11 first-grade indices, there was no consensus for one index (CV = 0.208 > 0.2). Five of the 48 s-grade indices were not agreed upon: a1 (CV = 0.208), a4 (CV = 0.208), c4 (CV = 0.223), d1 (CV = 0.238), and g1-2 (CV = 0.220). (Table 3).

**Table 3:** Results of phase 1 of importance and accuracy ratings of indices

First-grade Indices	Importance			Second-grade Indices	Accuracy		
	Mean	SD	CV		Mean	SD	CV
a	4.640	0.569	0.123	a1	4.040	0.841	0.208
				a2	4.320	0.690	0.160
				a3	4.520	0.586	0.130
				a4	4.040	0.841	0.208
b	4.640	0.569	0.123	b1	4.240	0.831	0.196
				b2	4.360	0.700	0.161
				b3	4.520	0.510	0.113
				b4	4.360	0.638	0.146
c	4.600	0.500	0.109	c1	4.400	0.646	0.147
				c2	4.360	0.700	0.161
				c3	4.440	0.768	0.173
				c4	4.080	0.909	0.223
d	4.600	0.646	0.140	d1	4.200	1.000	0.238
				d2	4.440	0.712	0.160
				d3	4.680	0.476	0.102
				d4	4.160	0.688	0.165
e	4.360	0.700	0.161	e1	4.320	0.748	0.173
				e2	4.360	0.757	0.174
				e3	4.560	0.712	0.156
				e4	4.160	0.746	0.179
f	4.320	0.900	0.208	f1	4.160	0.800	0.192
				f2	4.320	0.627	0.145
				f3	4.400	0.577	0.131
				f4	4.120	0.600	0.146
g	4.640	0.569	0.123	g1−1	4.440	0.583	0.131
				g2−1	4.520	0.510	0.113
				g3−1	4.640	0.490	0.106
				g4−1	4.280	0.737	0.172
				g1−2	4.040	0.889	0.220
				g2−2	4.400	0.577	0.131
				g3−2	4.600	0.577	0.126
				g4−2	4.240	0.663	0.156
h	4.680	0.476	0.102	h1	4.400	0.577	0.131
				h2	4.640	0.490	0.106
				h3	4.720	0.458	0.097
				h4	4.320	0.627	0.145
i	4.640	0.569	0.123	i1	4.360	0.700	0.161
				i2	4.440	0.583	0.131
				i3	4.600	0.646	0.140
				i4	4.280	0.678	0.158
j	4.800	0.408	0.085	j1	4.360	0.700	0.161
				j2	4.520	0.586	0.130
				j3	4.600	0.500	0.109
				j4	4.360	0.700	0.161
k	4.640	0.569	0.123	k1	4.360	0.638	0.146
				k2	4.480	0.586	0.131
				k3	4.440	0.712	0.160
				k4	4.440	0.651	0.147

Five experts also made specific suggestions: three experts thought that the indicator for social needs (k) should be changed to social services; one expert thought that the specific descriptions of the curriculum system (i) should be revised and improved in each cycle; and one expert thought that the secondary indices for research methods (c), c3 and c4, should be further differentiated. (Table 4).

**Table 4:** Expert opinion

Expert	Specific recommendations
No.8	Can additional social services be considered based on social needs (k)?
No.6	The subjects described in i1, i3, and i4 are *courses*, whereas the subject of i2 is *students*. i1, i2, i3, and i4 should be consistent in the subjects they describe to be comparable.
No.11	There are differences between basic and applied disciplines, such as more pronounced differences in social needs and personnel training.
No.12	Social service is the focus of the fifth round of disciplinary assessment in China, and social service is more important in disciplinary assessment relative to the need for meetings.
No.19	c3 and c4 should be further differentiated.

### Phase 2

Before starting phase 2 of the Delphi, the subject team adjusted the two primary indicators (f, k) and 10 secondary indicators (a1, a4, c4, d1, g1-2, i2, k1, k2, k3, k4) based on the comments of the experts and combined with the discussions of the group. (Table 5) The following are the specific reasons for the changes:

**Table 5:** Revision of indicators

No.	Phase 1	Phase 2
f	Degree granting	Number of degree-granting units
k	Social needs	Social service
a1	Field X focuses on a variety of specific problems of the same type	Field X focuses on specific individual issues
a4	Field X studies a class of problems with well-defined attributes, and the attributes of these problems are shifting	X field research content attributes are shifting
c4	Knowledge dissemination through various vehicles such as teaching materials, books, audio and video in field X	The number of various carriers, such as textbooks, books, audio, and video, in field X began to decline
d1	Research methods in field X are mainly qualitative and relatively homogenous	Research methods in field X are relatively homogenous
g1−2	There are only a few research grants in field X	There is almost no national research funding in field X
i2	There are instructional programs for graduate and doctoral students in Area X	Specialized courses of instruction begin in field X
k1	Society is not yet aware of the need for professionals in field X	Society is not yet aware of the need for specialized services in field X
k2	There is a social demand for people in field X	There is a social demand for services in field X
k3	Society has a steady and sizeable demand for talent in X field	Stable and sizeable social demand for services provided in field X
k4	Society’s demand for talent in field X has declined	Reduced social demand for services in field X

f. The secondary indicator reflects the number of degree-granting units. The primary level indicator “Degree-granting” is too broad.

k, k-1, k-2, k-3. China’s fifth disciplinary assessment places greater emphasis on social services. The more mature a discipline is, the more the community will demand the services of that discipline, not the higher the employment rate.

a1. According to Thomas S. Kuhn’s explanation of the new paradigm in The Structure of scientific revolutions, in the early stages of the formation of the new paradigm, the researcher is the one who discovers individual anomalies in the old paradigm [27]. The process of formation of a discipline is the process of paradigm generation, and therefore the object of study of a discipline in its infancy should be individual and specific problems.

a4. Remove redundancies and improve clarity of presentation.

c4. Nonaka categorizes knowledge into tacit and explicit [26]. Explicit knowledge, also known as coded knowledge, can be passed on and learned through carriers. Tacit knowledge, also known as tacit knowledge, exists only in the experiences and feelings of individuals. According to the theory of the disciplinary development cycle, the development process of disciplinary knowledge should be the process of changing from tacit knowledge to explicit knowledge, so in the decline phase, the transmission carriers of related knowledge should decrease.

d1. A more concise formulation.

g1-2. In China, a certain amount of scientific research is necessary to obtain support from national funds. The embryonic stage of the development of a discipline is a process of accumulating scientific results, so it is almost impossible to obtain national research funds at this stage.

i2. Educational programs for medical students should not only be found in postgraduate and doctoral education during the period of development of the discipline.

During the second phase, the response rate of the experts was 100%(25/25). The mean CV for indices importance was 0.115 and the mean CV for indices descriptive accuracy across cycles was 0.154. All first-grade indices and second-grade indices have a CV of less than 0.2.

### Credibility of panelists

The Ca and Cs coefficients of the expert panel were categorized as 0.892 and 0.716, and the Cr coefficient was 0.804, indicating that the members of the expert panel had a high degree of authority.

## Discussion

This MHMM is proposed primarily as a reference for decision-making by Chinese educational administrators. After two rounds of Delphi procedures, a maturity model of medical humanities based on life cycle theory was developed.

In China, the results of disciplinary assessment have become an important reference basis for resource allocation and disciplinary adjustment [28]. First-level disciplines are the targets for disciplinary assessment [29]. Thus the second-level disciplines receive limited resources. Restructuring a second-level discipline to a first-level discipline requires an institution-led initiative to apply to the national administration [30]. Currently, the maturity of a secondary discipline can only be judged empirically by the researcher [31]. And the disciplinary maturity assessment system proposed in this study provides an objective evaluation tool for disciplinary maturity evaluation.

In phase 1, three experts considered that the social services index should replace the social needs index. They believe that the more mature a discipline is, the more the community will demand the services of that discipline, not the higher the employment rate [32]. It is consistent with the focus of China’s fifth round of discipline assessment, which increasingly emphasizes the impact of disciplines on the real world [33]. For example, the Social Medicine and Health Care Administration program should translate policy research into policy promotion and implementation. Translation of results plays a more significant role in disciplinary assessment than research results.

This study establishes a disciplinary maturity evaluation system based on life cycle theory. The purpose is not to identify the most notable markers of each discipline across the cycles but to provide a reference. At the end of phase 2, there was general agreement among the experts on the secondary indices. Indicates that the experts consider the indicator’s reference markers to be more reasonable across cycles. In China, this is the first study focusing on the maturity assessment of second-level disciplines; therefore, it may be beneficial for further research on disciplinary research [34]. In the past, researchers in a particular field of study could only conduct disciplinary evaluations by collecting data on papers, monographs, etc., from all university researchers in the country [35]. This method requires a lot of money and takes a lot of time, and the process is complicated [36]. The maturity model of this study can provide a simple and efficient method for disciplinary evaluation of all research fields that do not belong to the first-level disciplines.

There is a growing need for medical humanities knowledge in the country, especially after experiencing COVID-19 [37, 38]. More medical professionals are focusing on medical humanism while treating diseases [39]. Medical schools and healthcare organizations are also focusing more on developing empathy in medical students and healthcare workers [40]. As a result, there has been a growing call for the medical humanities to be established as a first-level discipline. However, medical humanities is only a second-level discipline in China, with limited educational resources to train professionals and specialized knowledge that cannot be translated into reality. The MHMM model provides a scientific research tool for the restructuring medical humanities into a first-level discipline.

## Conclusion

In our study, a two-round Delphi method was used, and 25 experts reached a consensus on indicators for evaluating the maturity of the medical humanities, which can be applied to the assessment of secondary disciplines. This study also provides an essential research tool for upgrading medical humanities to a first-level discipline in China.

### Strengths and limitations

An essential limitation of this study is the comparison of the results of this study with nationally organized disciplinary assessments. Given the complexity of disciplinary assessment in China, the group was unable to compare the two results with limited resources.

Second, nearly half of the experts in this study were medical experts. While this ensures that the medical humanities maturity model indicators are more generalizable, it risks reducing their relevance.

## Data Availability

The datasets generated and analysed during the current study are available upon reasonable request with the corresponding author.
